# Highly Dispersed Ni on Nitrogen-Doped Carbon for Stable and Selective Hydrogen Generation from Gaseous Formic Acid

**DOI:** 10.3390/nano13030545

**Published:** 2023-01-29

**Authors:** Alina D. Nishchakova, Dmitri A. Bulushev, Svetlana V. Trubina, Olga A. Stonkus, Yury V. Shubin, Igor P. Asanov, Vladimir V. Kriventsov, Alexander V. Okotrub, Lyubov G. Bulusheva

**Affiliations:** 1 Nikolaev Institute of Inorganic Chemistry SB RAS, 3 Acad. Lavrentiev Ave., 630090 Novosibirsk, Russia; 2 Boreskov Institute of Catalysis SB RAS, 5 Acad. Lavrentiev Ave., 630090 Novosibirsk, Russia

**Keywords:** N-doped carbon, Ni single-atom catalyst, Ni clusters, Ni nanoparticles, formic acid, hydrogen production

## Abstract

Ni supported on N-doped carbon is rarely studied in traditional catalytic reactions. To fill this gap, we compared the structure of 1 and 6 wt% Ni species on porous N-free and N-doped carbon and their efficiency in hydrogen generation from gaseous formic acid. On the N-free carbon support, Ni formed nanoparticles with a mean size of 3.2 nm. N-doped carbon support contained Ni single-atoms stabilized by four pyridinic N atoms (N_4_-site) and sub-nanosized Ni clusters. Density functional theory calculations confirmed the clustering of Ni when the N_4_-sites were fully occupied. Kinetic studies revealed the same specific Ni mass-based reaction rate for single-atoms and clusters. The N-doped catalyst with 6 wt% of Ni showed higher selectivity in hydrogen production and did not lose activity as compared to the N-free 6 wt% Ni catalyst. The presented results can be used to develop stable Ni catalysts supported on N-doped carbon for various reactions.

## 1. Introduction

Hydrogen is a powerful clean fuel, but numerous technical problems prevent the creation of a large-scale hydrogen economy. The use of liquid organic hydrogen carriers allows elimination of some transportation and hydrogen storage problems. Among these carriers, formic acid could be preferred as hydrogen source due to high hydrogen content of 4.4 wt%, ability to be safely handled at room temperature, its nontoxicity and production from biomass [1,2] or by CO_2_ hydrogenation [3,4]. Various homogeneous and heterogeneous catalysts based predominantly on noble metals have been reported for the production of hydrogen from formic acid.

Nickel, as it has low cost, could be a suitable catalyst for different reactions including hydrogen production from formic acid decomposition. Earlier, a number of experimental and theoretical works were devoted to study the mechanism of the formic acid decomposition over bulk Ni and supported Ni nanoparticles [5,6,7]. Catalysts based on bulk metals are often inferior to state-of-the-art single-atom catalysts due to a different coordination sphere of the metal atom and changes in electronic properties [8,9,10,11,12]. As compared to bulk metals, all metal sites in single-atom catalysts are accessible by reactants. This may provide their efficient utilization. It is important that the rate and selectivity for some reactions taking place over single-atom catalysts can significantly exceed the rate over traditional supported catalysts, while maintaining high stability. This allows the reactions to be carried out at lower temperatures with less energy consumption.

Metal in a single-atom state needs stabilization on the surface of the support to avoid aggregation during the reaction [13,14], hence, the nature of the support is very important. Porous carbon materials possess a unique combination of accessible high surface area, thermal, mechanical and chemical stability, and are widely used in the processes of sorption and catalysis [15,16]. These materials are often synthesized by carbonization of carbon-containing compounds, frequently having a natural origin, such as biomass wastes. In such case, the preparation of a raw material (separation, crushing, etc.) is needed before the direct carbonization. However, this method does not allow proper control of the morphology, surface area and porosity of the product [17]. These parameters can be more fine-tuned using template synthesis [18], and, in particular, template-assisted chemical vapor deposition (CVD) [19]. A variety of templates, precursors and synthesis conditions allow the tuning of the structure of the resulting material [20] and the insertion of non-carbon atoms [21]. Nitrogen is the most widely used for insertion into the carbon structure [22]. The concentration and chemical state of nitrogen introduced into the carbon material depend on doping conditions [23], especially temperature [21]. Many studies have already reported that nitrogen in a carbon support may stabilize metal in the form of single atoms [12,24].

Among the literature data attributed to Ni single-atom catalysts, there are few works discussing metal loading of more than 5 wt%. Ni atoms were reported to be coordinated to 2–5 nitrogen atoms and Ni content ranged from 5.3 to 7.5 wt% [25,26,27], although there are a couple of studies with Ni loading of 18–20 wt% [28,29], anchored on nitrogen-doped carbon nanotubes. Despite synthesizing Ni single-atom catalysts with outstanding metal content, none of these works tested them in traditional hydrogen production reactions. Previously, we reported [30] about 1 wt% Ni catalysts on N-doped and N-free porous carbon supports, in which Ni was in a single-atom state in the former case and in a state of nanoparticles with a mean size of 3.9 nm in the latter case. Briefly, in the N-doped catalyst, Ni atoms were presented in the Ni^2+^ state coordinated by four pyridinic nitrogen atoms (Ni-N_4_-sites). The apparent activation energy of hydrogen generation from gaseous formic acid was 110 kJ mol^−1^ for the single-atom catalyst vs 99 kJ mol^−1^ for the catalyst with nanoparticles. Density functional theory (DFT) calculations led to a possible reaction mechanism, in which the highest energy barrier, the hydrogen recombination step, was close to the experimental apparent energy of the reaction for the single-atom catalyst. 

The field of single-atom catalysts is a rapidly developing area. However, it is still unclear what happens when the content of active metal exceeds the content of the support sites that can stabilize these single atoms. Recently, Lepre et al. observed the formation of sub-nanosized Ni clusters on the nitrogen-doped carbonaceous support [31], but no explanation was given for the mechanism of Ni clustering. The presented study covers this area. 

In this work, 6 wt% nickel is deposited on carbon (C) and N-doped carbon (CN) by impregnation of pre-synthesized 1Ni/C and 1Ni/CN samples (1Ni corresponds to 1 wt% metal content) with the required amount of Ni acetate, followed by reduction. This procedure allows discrimination of the effect of the Ni-N_4_-sites present in 1Ni/CN on further metal deposition. The obtained samples are used as catalysts for the gas-phase decomposition of formic acid. The structure and composition of the samples are studied after the reaction. X-ray diffraction (XRD), high-angle annular dark field scanning transmission electron microscopy (HAADF/STEM) and X-ray photoelectron spectroscopy (XPS) revealed Ni nanoparticles in the 6Ni/C and sub-nanosized Ni species in the 6Ni/CN samples. DFT calculations showed that, after the formation of single-atom Ni-N_4_-sites, newly arriving Ni atoms prefer to be located near nitrogen atoms rather than in the regions of N-free carbon. They form small Ni clusters, detectable by extended X-ray absorption fine structure (EXAFS) spectroscopy. Catalytic tests showed that Ni single-atoms and Ni sub-nanoclusters on the CN support possess very similar activity in the process of hydrogen generation and higher stability and selectivity as compared to Ni nanoparticles on the C support.

## 2. Results

### 2.1. Structural Characterization 

**Figure 1:**
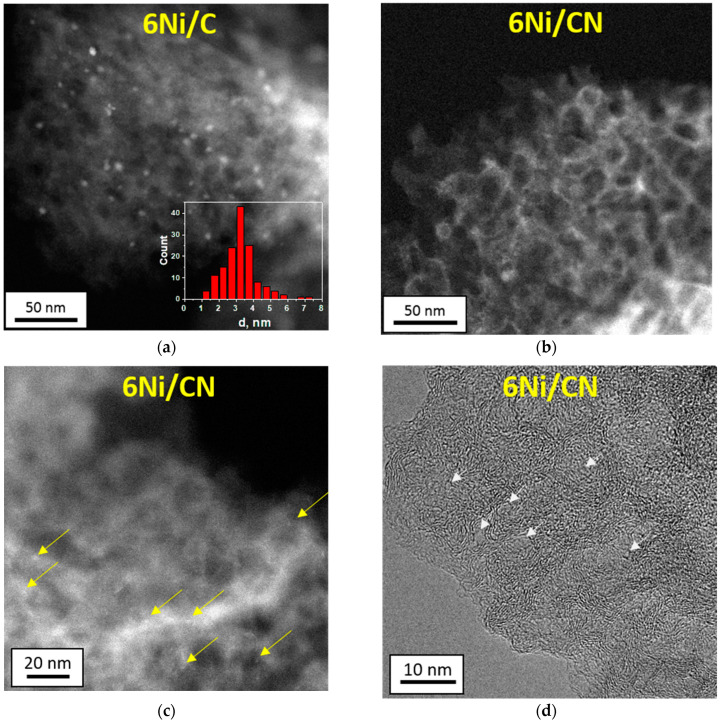
HAADF/STEM low-magnification images of (**a**) 6Ni/C and (**b**) 6Ni/CN and (**c**) high-magnification image of 6Ni/CN. Inset in (**a**) presents the size distribution of nanoparticles. Yellow arrows in (**c**) indicate Ni sub-nanospecies. (**d**) High-resolution TEM image of the 6Ni/CN nanomaterial. White arrows show pores in the CN nanomaterial.

The HAADF/STEM image of the 6Ni/C sample showed the presence of many round-shape nanoparticles (Figure 1a), which should be attributed to nickel because this element is heavier than carbon and, therefore, it scatters more electrons. The mean size of Ni nanoparticles determined from the distribution inserted into Figure 1a is about 3.2 nm. Interestingly, the mean size of Ni nanoparticles in 1Ni/C, used as a support in the synthesis of 6Ni/C, is 3.9 nm (Figure S1). Therefore, the nickel present in the support affects the decomposition of nickel acetate and, as a consequence, the size of the formed Ni nanoparticles. TEM study confirms the presence of nanoparticles in the 6Ni/C sample (Figure S2a). 

**Figure 2:**
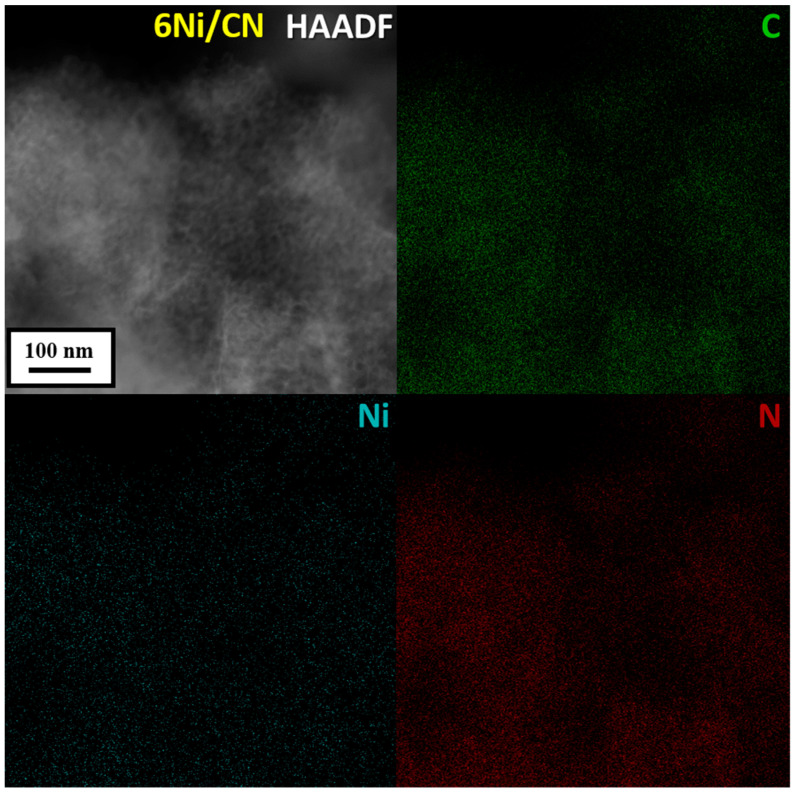
HAADF/STEM image and corresponding elemental mapping of C, Ni and N in the 6Ni/CN nanomaterial after the catalytic reaction.

HAADF/STEM does not detect Ni nanoparticles in 6Ni/CN (Figure 1b). However, the image at higher magnification shows bright spots smaller than 1 nm in size (Figure 1c), which can be attributed to nickel. Nanoparticles are also not visible when examining 6Ni/CN using TEM (Figure S2b). At higher magnification, the layered structure of the carbon support is clearly revealed (Figure 1d). The highly curved intertwining layers do not make it possible to detect the presence of sub-nanosized nickel species on the substrate. This was done using energy dispersive X-ray (EDX) spectroscopy. Elemental mapping of the 6Ni/CN sample shows a uniform surface distribution of the main elements, including Ni (Figure 2). Ni lines are also present in the EDX spectrum of the sample (Figure S3). Ni species observed in Figure 1c are probably the largest in the 6Ni/CN samples, while most of the nickel remains invisible in current imaging conditions. 

**Figure 3:**
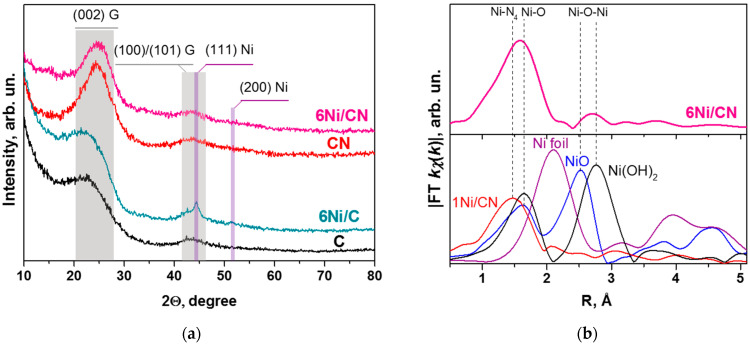
(**a**) XRD patterns of N-free (C) and N-doped (CN) supports and 6Ni/C and 6Ni/CN nanomaterials after the catalytic reaction. G denotes graphite. (**b**) The K-weighted Fourier transform spectrum of 6Ni/CN catalyst after the reaction (top panel) and spectra of 1Ni/CN, Ni foil, NiO, and Ni(OH)_2_ (bottom panel) without taking into account the phase shift.

The absence of Ni nanocrystals in the 6Ni/CN sample is confirmed by XRD study (Figure 3a). To elucidate reflections related to nickel containing particles, the patterns of the supports were also recorded. They exhibited two wide reflections highlighted by grey area and centered at 2Ɵ = 24° and 43° that are attributed to the (002) and (100)/(101) graphite planes, respectively. These reflections are preserved in the XRD patterns of the 6Ni/CN and 6Ni/C samples. Additional peaks at 2Ɵ = 44.5° and 51.5°, attributed to the (111) and (200) reflections of metallic Ni, respectively, appear in the 6Ni/C pattern. The mean size of Ni particles estimated from the width at half height of the reflections is 4.5 nm. This estimation is rough due to overlapping of the (111) Ni reflection with the (100)/(101) graphite reflections. The absence of Ni related reflections in the XRD pattern of 6Ni/CN indicates a very high dispersion of Ni in this sample.

The EXAFS study was carried out to elucidate the local environment of Ni in 6Ni/CN. The first peak of the radial distribution function for 6Ni/CN is shifted towards longer distances as compared to that in the 1Ni/CN sample, where Ni atoms are bonded with nitrogen atoms (Figure 3b). The shift may be associated with the coordination of some of Ni atoms with more electronegative elements, in particular, with oxygen. However, the peaks in the reference spectra of NiO and Ni(OH)_2_ are located at longer distances than those in the 6Ni/CN spectrum. 

The 1Ni/CN sample was used as a support to synthesize 6Ni/CN, therefore the latter must contain Ni-N_4_ centers, where Ni atom is bonded to four N atoms. This coordination was fixed in modeling the EXAFS data and the results are summarized in Table 1. The fraction of the Ni-N_4_ centers in 6Ni/CN is 30% and the rest of nickel is surrounded by about 5.5 oxygen atoms. The Ni–O distance is about 2.09 Å. The second peak in the 6Ni/CN spectrum corresponds to Ni–O–Ni bonding with the average distance of 3.11 Å and a coordination number of 0.8, which indicates that the number of Ni atoms in the clusters does not exceed several atoms. The simulation results are consistent with the HAADF/STEM (Figure 1b,c) and XRD (Figure 3a) data. 

### 2.2. Electronic State of Elements

The surface concentration of elements in the 6Ni/C and 6Ni/CN nanomaterials, determined from XPS data, is presented in Table 2. The higher concentration of Ni in 6Ni/CN confirms the fine dispersion of the metal on the CN support. The coordination of a part of the Ni atoms with oxygen, as shown by the EXAFS data, is the reason for the larger oxygen content in 6Ni/CN as compared to 6Ni/C. 

**Figure 4:**
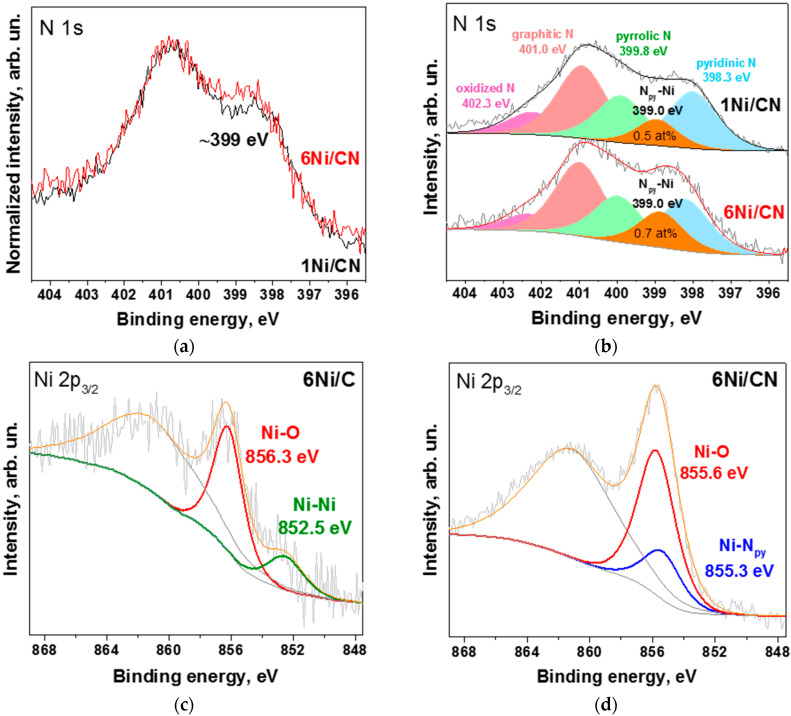
XPS N 1s spectra of 1Ni/CN and 6Ni/CN: (**a**) normalized and (**b**) fitted by five components; XPS Ni 2p_3/2_ spectra of (**c**) 6Ni/C and (**d**) 6Ni/CN samples. All measurements were performed after the catalytic reaction.

A direct comparison of the N 1s spectra of the initial 1Ni/CN sample and the 6Ni/CN sample shows an increase in the intensity of the latter spectrum at ~399 eV (Figure 4a). The N 1s spectra of both samples are fitted by five components (Figure 4b), corresponding to pyridinic N (N_py_, 398.3 eV), N_py_-Ni bonds (399 eV), pyrrolic N (399.8 eV), graphitic N (401.0 eV) and oxidized N species (402.3 eV) [26,32]. An analysis of the N 1s spectra gives the content of nitrogen involved in the N_py_-Ni bonds as ~0.5 at% for 1Ni/CN and ~0.7 at% for 6Ni/CN. Thus, for the 6Ni/CN sample, the fraction of N_py_-Ni species is 24%, which agrees well with the EXAFS fitting data (30% in Table 1). 

The XPS Ni 2p_3/2_ line of 6Ni/C showed a weak peak at 852.5 eV attributed to metallic Ni (Figure 4c). The dominant peak at 856.3 eV corresponds to nickel oxide or hydroxide species [33,34]. Therefore, on the N-free carbon support, nickel is in the form of nanoparticles, the surface of which is oxidized due to the contact with oxygen/moisture in the air [35]. 

The XPS Ni 2p_3/2_ line of 6Ni/CN was fitted by two components (Figure 4d) since two different nickel configurations were detected by EXAFS spectroscopy (Figure 3b). The Ni-N_py_ component at 855.3 eV comes from Ni-N_4_ centers [30,36] and the Ni-O component at 855.6 eV corresponds to sub-nanometric oxidized Ni clusters [37]. The resulting ratio of the two states of nickel (75% of Ni-O and 25% of Ni-N_py_) is in a good agreement with the EXAFS data (Table 1).

**Figure 5:**
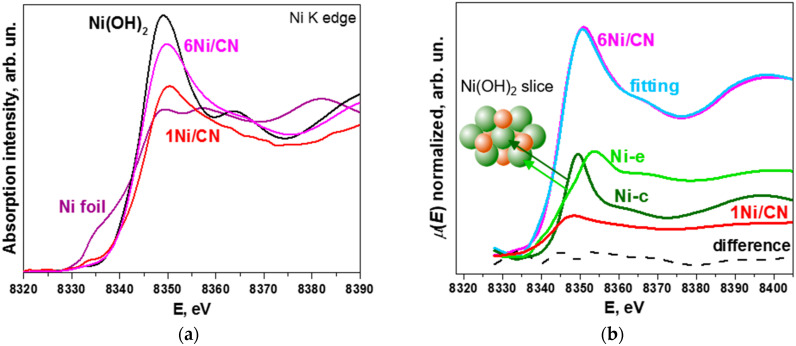
(**a**) XANES Ni K-edge spectra of 1Ni/CN and 6Ni/CN in comparison with Ni foil and Ni(OH)_2_ reference spectra. The measurements were performed on samples after the catalytic reaction. (**b**) The fitting of the experimental Ni K-edge spectra of 6Ni/CN using a linear combination of the experimental spectrum of 1Ni/CN and the spectra simulated for the central Ni atom (Ni-c) and edge Ni atom (Ni-e) in Ni(OH)_2_. Dashed curve is the difference between experimental and synthetic spectra. Inset in (**b**) shows Ni(OH)_2_ slice consisting of 7 Ni atoms (green) and 6 O atoms (red).

Figure 5a compares X-ray absorption near-edge structure (XANES) Ni K-edge spectra of 1Ni/CN and 6Ni/CN with Ni foil and Ni(OH)_2_ references. The spectra of the catalysts exhibit no pre-edge step corresponding to the presence of metallic Ni, which is seen in the spectrum of Ni foil. This confirms that the N-doped support inhibits the formation of Ni nanoparticles. The white line for 6Ni/CN is significantly increased as compared to that for the 1Ni/CN sample, indicating that Ni is oxidized, but still not as strongly as in Ni(OH)_2_. Since the structural data for 6Ni/CN obtained from the EXAFS Ni K-edge spectrum fitting (Table 1) reveal Ni-N_4_ sites and small Ni oxidized clusters, the reason may lay in the difference in the electronic state of nickel in bulk Ni(OH)_2_ and small Ni(OH)_2_ clusters.

Three components are taken to describe the XANES Ni K-edge spectrum of 6Ni/CN (Figure 5b). The first component is the experimental XANES Ni K-edge spectrum of the 1Ni/CN sample, which was used as support in the synthesis. The second and the third components are the XANES spectra simulated for the central Ni atom (Ni-c) and the edge Ni atom (Ni-e) of the Ni(OH)_2_ slice (Figure 5b). The proportions of the components are listed in Table 3. The difference between the experimental 6Ni/CN spectrum and the synthetic spectrum is negligible (dashed line in Figure 5b). The intensity of the 1Ni/CN spectrum is ~25% in the fitting of the XANES Ni K-edge spectra of 6Ni/CN. This value well agrees with amounts of Ni-N_4_ centers determined from XPS and EXAFS data.

### 2.3. Catalytic Properties 

**Figure 6:**
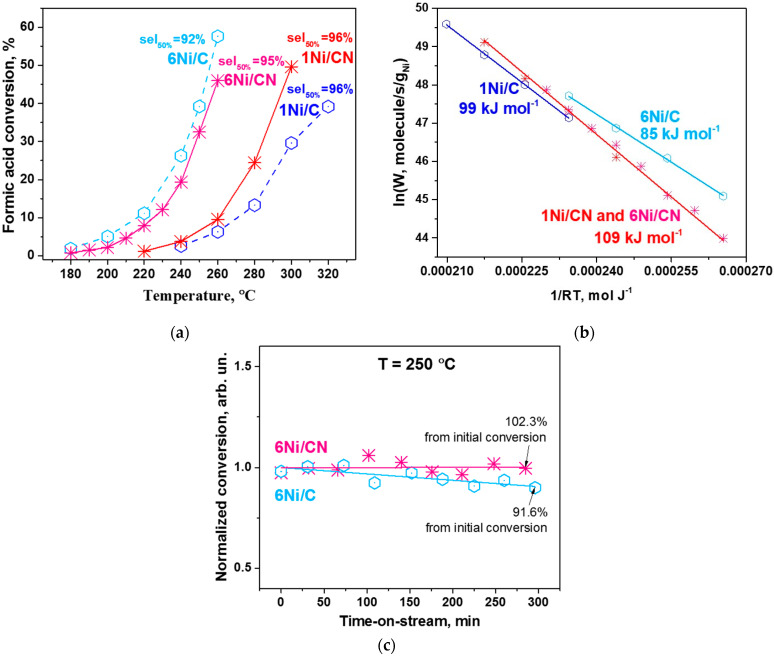
(**a**) Conversion-temperature dependences and (**b**) Ni mass-based Arrhenius plots for 1Ni/C, 1Ni/CN, 6Ni/C and 6Ni/CN (7 mg each). The selectivity to H_2_ at 50% of formic acid conversion of each catalyst is shown in (**a**), the apparent activation energy with an error of ±2 kJ mol^−1^ is indicated in (**b**). (**c**) Stability of 6Ni/C (3.1 mg) and 6Ni/CN (3.3 mg) in the reaction at 250 °C during 5 h tests. The ratio of the final to the initial conversion is indicated.

The catalytic properties of the obtained 6 wt% catalysts on N-free and N-doped porous carbon materials were tested in the gas-phase formic acid decomposition reaction and compared to the corresponding results of previously studied 1 wt% Ni catalysts [30]. Figure 6a shows the formic acid conversions for all Ni catalysts tested. Although 1Ni/CN shows a higher formic acid conversion with increasing reaction temperature as compared to the N-free 1Ni/C catalyst, the highly dispersed N-containing 6Ni/CN catalyst demonstrates conversion close to that of 6Ni/C with Ni nanoparticles at temperatures lower than 220 °C. However, the selectivity of the N-doped catalysts with respect to the H_2_ production remains practically unchanged (95–96%) with increase in the nickel content.

The specific Ni mass-based reaction rates for all samples were calculated and presented as Arrhenius plots in Figure 6b. It is seen that, for the N-doped catalysts, the Ni concentration does not affect the rate, so all data can be presented in one Arrhenius line (red line). The results show that the calculated values of apparent activation energy for the N-doped and N-free catalysts are different. For N-doped catalysts, the energy is higher and corresponds to 109 kJ mol^−1^, which indicates that the nature and mass-based concentration of active Ni in these catalysts remain the same. For N-free catalysts, the apparent activation energies are generally lower.

An important parameter demonstrating the performance of catalysts is their stability in hydrogen production. Previously, we have shown good stability of the single-atom 1Ni/CN catalyst in the formic acid decomposition reaction [38]. Herein, we examined the stability of the 6Ni/CN catalyst and compared it with the 6Ni/C catalyst at 250 °C (Figure 6c). The test shows that this N-doped catalyst is also very stable. In contrast, the catalyst containing Ni nanoparticles loses its activity by 8.4% after 5 h. 

## 3. Discussion

**Figure 7:**
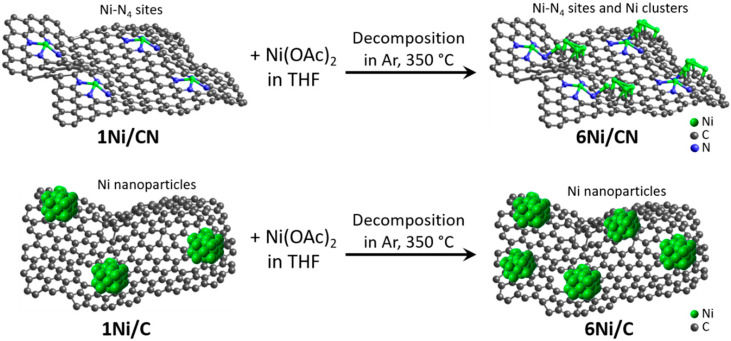
Schematic representation of layers in 1Ni/C and 1Ni/CN nanomaterials, illustration of 6Ni/C and 6Ni/CN synthesis and nickel distribution (green balls) in all samples.

In this work, to obtain the catalysts with supported Ni content of 6 wt%, 1Ni/C and 1Ni/CN nanomaterials are impregnated with a solution of Ni acetate in tetrahydrofuran (THF) (Figure 7). Structural study of these original nanomaterials revealed Ni nanoparticles 3.9 nm in size in 1Ni/C and single Ni atoms stabilized by four N atoms of a double vacancy in 1Ni/CN [30]. The C and CN layers are not flat (Figure 1 and Figure S2) because they were templated by CaO nanoparticles. The growing layers envelop the templates and join in the space between adjacent nanoparticles. The Raman spectra of the 1Ni/C and 1Ni/CN samples revealed a G-band at 1595 cm^−1^ from sp^2^ carbon and a D-band at 1362 cm^−1^ (Figure S4), activated by defects present in a honeycomb carbon lattice. Thus, the layers in 1Ni/C and 1Ni/CN can be represented as a curved sp^2^-hybridized carbon network with many defects (Figure 7). The deposited nickel acetate molecules decompose in an argon atmosphere at 350 °C to form 6Ni/C and 6Ni/CN. The intensity ratios of the D to G peaks in the Raman spectra of these nanomaterials do not change as compared to the values for the initial counterparts (Figure S4). Consequently, no additional defects appear in the C and CN layers during deposition. 

6Ni/C and 6Ni/CN were used as catalysts in the reaction of hydrogen generation from gaseous formic acid. It was found that both catalysts prepared on N-free carbon support (1Ni/C and 6Ni/C) show the apparent activation energy lower than that for the catalysts on N-doped carbon (Figure 6b). At low reaction temperatures, the 6Ni/C catalyst exhibited increased mass-based catalytic activity as compared to the 1Ni/C catalyst, while no difference was found between the N-doped 6Ni/CN and 1Ni/CN catalysts.

It is obvious that the support affects the structure of Ni active centers. Therefore, we invoked a set of methods to characterize nanomaterials after the catalytic reaction. 

According to the HAADF/STEM (Figure 1a), XRD (Figure 3a) and XPS (Figure 4c) data, the 6Ni/C catalyst contains surface-oxidized Ni nanoparticles with an average size of 3.2 nm. Interestingly, the addition of Ni to initial 1Ni/C leads to an increase in the number of nanoparticles on carbon support, but not to an increase in their size (Figure 7). The oxidized surface of Ni nanoparticles in 1Ni/C probably hinders their growth upon the addition of a new portion of Ni acetate and the nuclei of Ni nanoparticles occupy the free carbon surface. 

The above characterization methods found no signs of Ni nanoparticles in the 6Ni/CN catalyst. However, the EXAFS/XANES examination of this sample revealed the presence of Ni–O–Ni bonds (Figure 3b and Figure 5b). These bonds are formed upon the contact of Ni clusters with oxygen-containing molecules present in air. In the reducing atmosphere of the catalytic reaction, oxidized Ni transforms into the metallic state. Simulation of the XANES Ni K-edge spectrum detects that the fraction of edge Ni atoms in oxidized Ni species is larger than that of central Ni atoms (Table 3), so the clusters are rather small (Figure 7). 

To understand why Ni atoms form clusters rather than nanoparticles on the 1Ni/CN surface, we used DFT calculations. The CN surface model was proposed based on XPS and EXAFS/XANES data. The XPS N 1s spectrum of 1Ni/CN revealed a component corresponding to the Ni bond to pyridinic N (Figure 4b) and the EXAFS study identified four equivalent Ni-N bonds 2.00 ± 0.02 Å long [30]. The interaction of a Ni atom with four pyridinic N atoms located at the edge of a double vacancy in a graphene network can provide the desired configuration. The DFT calculation of a flat N-doped graphene fragment with the attached nickel atom gives a Ni-N bond length of ~1.87 Å. This value agrees with the literature data, where the Ni-N bond in flat Ni-N_4_ configurations is always shorter than ~1.90 Å, regardless of the calculation method [39,40,41]. In addition, the corresponding value determined experimentally for nickel porphyrin is 1.951(2) Å [42], which is the limit for the planar Ni-N_4_ unit in sp^2^-hybridized carbon. However, the DFT calculation of the model with a strongly distorted octahedral coordination of the nickel atom to the pyridinic nitrogen atoms gave the Ni-N distances from 1.91 to 2.08 Å [27]. The model was proposed based on a comparison of the theoretical XANES Ni K-edge spectrum with the experimental one. This approach was applied to select the model of the 1Ni/CN nanomaterial used as a support in the synthesis of 6Ni/CN. 

The spectrum of a fragment of the curved carbon surface, which provides distorted Ni-N_4_ coordination, showed good agreement with the experimental Ni K-edge spectrum (Figure S5). A sphere diameter required to provide such distortion of the Ni-N_4_ unit is ~4.4 nm, which is in reasonable agreement with sizes of the pores seen in the high-resolution TEM image of the 6Ni/CN nanomaterial (shown by white arrows in Figure 1d). The convex surface (Figure S5c) can be a cap of a carbon nanotube or a fragment of a fullerene-like structure. The latter is more suitable in our case since the nanomaterials are porous. We built a Ni-N_4_C_78_ cage model that maintains the required local curvature around the embedded Ni atom while optimizing the geometry. The resulting average Ni-N distance is 2.049 Å, which is in good agreement with the EXAFS data. In the model, there is enough area to allow additional nickel atoms to move across the surface.

**Figure 8:**
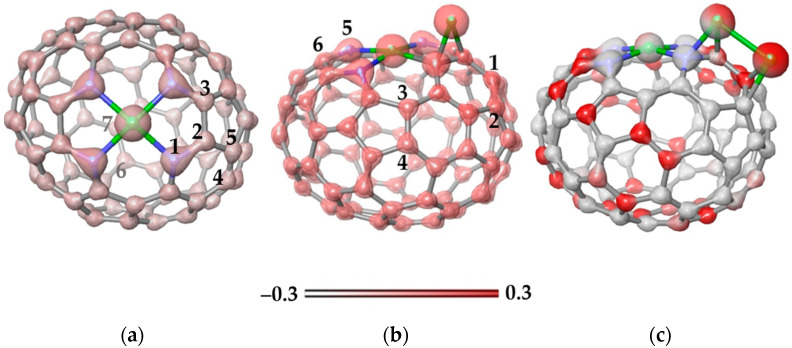
Electron density maps (iso-value of 0.3 kcal mol^−1^) plotted for (**a**) the Ni-N_4_C_78_ model and energetically preferable configurations of this model with (**b**) one attached Ni atom and (**c**) two attached Ni atoms. Optimized structures correspond to the ground state (triplet) of the models. N and Ni atoms are shown by blue and green balls, respectively. The numbers in (**a**,**b**) indicate the positions to add the next Ni atom. The energy gain from Ni atom addition is 1.914 eV for structure (**b**) and 2.100 eV for structure (**c**). The scale corresponds to the electrostatic potential.

Figure 8a shows the electron density map for the Ni-N_4_C_78_ model. An excess of electron density is observed around the N atoms, while the density is evenly distributed over the carbon atoms of the cage. The charge calculated using natural bond orbital (NBO) analysis is +0.70e for the Ni atom and −0.50e for each surrounding N atoms. The next Ni atom was located above the N–C bond (position 1–2 in Figure 8a) or over C–C bonds at positions 2–3, 4–5, and 6–7. According to calculations, the first position is the most energetically preferable due to the enhanced electron density on the N–C bonds (Figure 8a). It is important that the binding energy of 1.914 eV for the addition of Ni at this position is higher than the value of 1.256 eV for the addition of a Ni atom to the C–C bond in N-free carbon area (position 6–7). This indicates that the sites at the N atoms are more attractive to Ni atoms than a curved, non-functionalized carbon surface. 

The location of the Ni atom above the N–C bond causes a redistribution of the electron density (Figure 8b). In particular, an excess of density appears on the C–C bond (position 1–2) opposite the bond with Ni. The NBO charge on the new Ni atom is +0.23e, which is noticeably less than the charge of Ni in the Ni-N_4_ site of +0.75e. Attachment of the second Ni atom to the bonds labeled in Figure 8b showed that the position 1–2 is energetically more stable than the other considered cases (the bonds for attaching the Ni atom are numbered in Figure 8b). Two Ni neighbors form a bond (Figure 8c) and each atom donates about 0.41e to the carbon support. This electron density is evenly distributed over all atoms of the cage.

**Figure 9:**
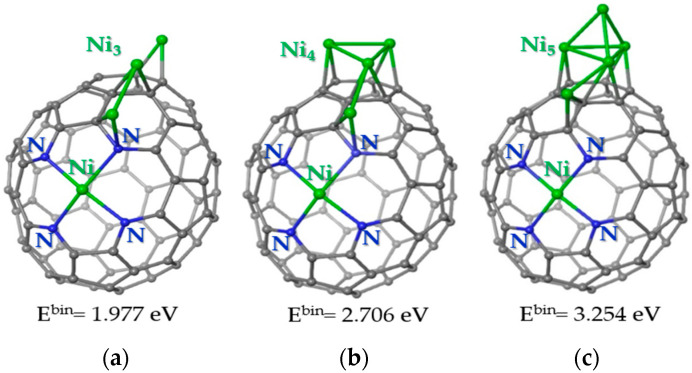
Energetically preferable locations of (**a**) three Ni atoms, (**b**) four Ni atoms, and (**c**) five Ni atoms on the carbon cage with the Ni-N_4_ site. Carbon, nitrogen and nickel are shown by grey, blue and green balls, respectively. The values correspond to the binding energy for the newly arrived Ni atom. A positive value indicates an energy gain. The ground state is triplet for structure (**a**) and quintet for structures (**b**,**c**).

The following calculations showed that the third Ni atom prefers to be located near the Ni-Ni pair (Figure 9a). In this case, the atom does not form Ni-Ni bonds with already deposited atoms. The lower binding energy as compared to the previous model (Figure 8c) indicates that the nickel atoms prefer to cluster on the carbon support. Indeed, four Ni atoms form a two-dimensional cluster near the Ni-N_4_ site (Figure 9b). Calculations show that the separation of two Ni-Ni pairs on the carbon cage is less favorable by 0.921 eV. The fifth Ni atom prefers to attach to the Ni surface, forming a three-dimensional Ni cluster (Figure 9c). Adding a Ni atom to an N-doped carbon surface requires much more energy. Consequently, at a certain size, the Ni cluster begins to grow vertically relative to the carbon support. 

The calculation results show that Ni-N_4_ sites play a decisive role in the formation of Ni clusters on the N-doped carbon surface. The EXAFS study of the initial 1Ni/CN support determines only the Ni-N bonds. According to the analysis of XPS N 1s spectra, the fraction of these bonds increases in the 6Ni/CN nanomaterial (Figure 4b). Consequently, with an increase in the Ni loading, N-terminated vacancies are occupied first. This result differs from the literate data, where the impregnation of an N,O-functionalized carbon support with nickel (II) acetylacetonate followed by calcination in air did not cause the bonding of nickel with nitrogen [31]. However, the nitrogen atoms stabilized nickel which allowed about 11 wt% of the nickel to be highly dispersed on the support. We confirm this role for the pyridinic N. When all of these nitrogen atoms are directly coordinated with nickel, they promote the deposition of nickel in their immediate vicinity due to the enhanced electron density in this location (Figure 8). The Ni-N_4_ site can initiate the growth of Ni clusters at each of the four nitrogen atoms. A sufficiently large number of such sites explains the small size of the clusters in the synthesized 6Ni/CN, despite the fact that the binding energy of the Ni atom increases with the size of the preformed Ni cluster (Figure 9). 

The most intriguing result of catalytic experiments is the same apparent activation energies for 1Ni/CN and 6Ni/CN (Figure 6b). The Ni atoms in 1Ni/CN are in the Ni-N_4_ sites and DFT calculations of the decomposition of formic acid molecule at this site showed that the recombination of the hydrogen atoms bonded to the Ni atom and the N atom requires the highest energy. In all found stable models with Ni deposited on Ni-N_4_C_78_, there is an atom close to the pyridinic N of the cage (Figure 8 and Figure 9), therefore, we expect the same reaction path for the 6Ni/CN catalyst as in the case of the 1Ni/CN catalyst. Note that DFT calculations predict the formation of a Ni-Ni pair with the second nickel in a vertical position to the planar Ni-N_4_ unit [43]. Herein, the clustering of nickel around the Ni-N_4_ sites is provided by the curvature of the CN supporting layers, which allows individual Ni-N_4_ sites to participate in the reaction.

The lower apparent energies observed for the 1Ni/C and 6Ni/C catalysts (Figure 6b) are provided by supported Ni nanoparticles. The (111) Ni reflection dominates in the 6Ni/C XRD pattern (Figure 3a). According to the DFT calculations, the highest energy barrier for the decomposition of formic acid on the Ni(111) surface is 99 kJ mol^−1^ [7,44], which is close to our experimental values. 

Although 6Ni/CN is less active than 6Ni/C in the gas phase decomposition of formic acid, this catalyst has a higher H_2_ selectivity (95%) (Figure 6a) and a much better stability (Figure 6c). The 6Ni/C catalyst during the 5-h test showed a decrease in conversion by 8.4%. Such behavior may be due to the sintering of nickel into nanoparticles under reaction conditions, previously reported for Ni catalyst [45,46,47]. Catalyst stability, especially at high temperatures, is an important parameter for any catalytic reaction, including hydrogenation reactions. 

The use of Ni single atoms for the catalytic generation of hydrogen from formic acid has so far been rarely reported; however, they are active in reactions involving different hydrogen donors. Feng et al. reported high activity and selectivity in the reduction of 5-hydroxymethylfurfural to 2,5-dihydroxymethylfuran at the Ni-N_4_ site in the presence of ethanol [48]. Zhang et al. effectively hydrogenated nitrobenzene to azoxybenzene using 2-propanol as a source of hydrogen and Ni_1_N_4_/NC as a catalyst [49]. Dai et al. reported the formation of a Ni-N_4_ site that showed higher activity and selectivity than Pd-based catalysts in acetylene hydrogenation using H_2_ [50].

The outstanding catalytic behavior of the Ni-N_x_ sites in hydrogen formation reactions is due to the excellent synergistic activity of neighboring nickel and nitrogen atoms. The developed 6Ni/CN catalyst with Ni-N_4_ sites and Ni clusters near them could be considered promising for these reactions. In our work, Ni with a content of 6 wt% was highly dispersed on N-doped carbon by a simple impregnation method. According to the literature data over the last 5 years [30,31,51,52], Ni in a content of ≥5 wt% is rarely synthesized in a highly dispersed state using the impregnation synthesis method. The co-assisted impregnation that utilizes additional stabilizing compounds such as 1,10-phenantroline [53] and Jacobsen’s ligand [54] to chelate Ni^2+^ ions provides a higher Ni concentration of up to 5.3 wt% [25]. However, a correctly selected and easily and cheaply produced support made it possible to achieve a high content (6 wt%) of highly dispersed Ni without additional stages of metal stabilization.

## 4. Conclusions

Nanomaterials 6Ni/C and 6Ni/CN containing 6 wt% Ni on carbon and N-doped carbon supports were synthesized by impregnating 1Ni/C and 1Ni/CN with a solution of Ni acetate in THF followed by annealing in an argon flow. The initial 1Ni/C and 1Ni/CN nanomaterials contained Ni in the form of nanoparticles about 3–4 nm in size and single Ni atoms stabilized by four pyridinic nitrogen atoms (Ni-N_4_ sites), respectively. According to the HAADF/STEM, XPS and XANES/EXAFS studies, the increase in Ni loading leads to a denser population of the carbon support with Ni nanoparticles and the appearance of small Ni clusters on the N-doped support. DFT studies showed that Ni clusters form near the Ni-N_4_ sites. A study of the decomposition kinetics of gaseous formic acid found the same apparent activation energy for 1Ni/CN and 6Ni/CN, which indicates that the reaction is determined by the same active site, involving Ni and neighboring N atoms. The 6Ni/CN catalyst demonstrated a slightly lower activity in the range of 180–320 °C as compared to the 6Ni/C catalyst; however, it showed a higher selectivity for the hydrogen production and a greater stability in the reaction. This is due to the high dispersion and strong bonding of nickel with the N-doped support. Our study shows that for further development of nickel single-atom catalysts, additional selection of easily synthesized supports containing a large number of N_4_ sites is promising. An increase in the amount of highly dispersed nickel can lead both to an increase in the efficiency of catalytic hydrogen production using the considered reaction and for hydrogenation and dehydrogenation reactions.

## 5. Materials and Methods

### 5.1. Synthesis of the Samples

Porous carbon (C) and N-doped carbon (CN) were synthesized in a horizontal tubular quartz reactor by decomposition of ethanol and acetonitrile vapors, respectively, at 800 °C on a CaO nanoparticle template. Details of the synthesis procedure are reported elsewhere [32]. Initially, 1Ni/C and 1Ni/CN nanomaterials were prepared by impregnation of C and CN with a Ni acetate solution in THF [30]. These nanomaterials were used for synthesis of 6Ni/C and 6Ni/CN. For this, 45.1 mg of Ni(OAc)_2_·4H_2_O was dissolved in THF with stirring for 20 min at room temperature, then 200 mg of 1Ni/C or 1Ni/CN were added and the mixture was stirred for 4 h at 60 °C. The enhanced temperature was chosen for faster dissolution of Ni(OAc)_2_ in THF and removal of excess of solvent molecules during the synthesis. To decompose the supported nickel acetate, the obtained materials were introduced into a reactor preliminary heated to 350 °C in an Ar flow for 30 min and then immediately cooled to room temperature without access to air.

### 5.2. Characterization Methods

Before any characterization, the nanomaterials were treated in the reductive atmosphere, namely in a flow of 2.5 vol% formic acid in Ar, at 350 °C for 30 min. XRD patterns were taken on a Shimadzu XRD-7000 diffractometer (Shimadzu Europa GmbH, Duisburg, Germany) using Cu Kα radiation and Ni filter on the reflected beam. To identify the phases, the position and intensity of the reflections were compared with the data from the JCPDS-PDF database. The high-resolution TEM images were obtained using a JEM-2200FS microscope (JEOL Ltd., Tokyo, Japan) with a Cs-corrector operated at 200 kV. The elemental mapping was carried out on a double-corrected transmission electron microscope Themis Z (Thermo Fisher Scientific, Waltham, MA, USA) with an accelerating voltage of 200 kV and a limit resolution of 0.06 nm in a HAADF/STEM mode using a Ceta 16 CCD matrix. Raman spectra were measured on a LabRAM HR Evolution (Horiba, Kyoto, Japan) spectrometer using a 514 nm excitation of an argon laser. XPS measurements were conducted on a SpecsLab PHOIBOS 150 spectrometer (SPECS GmbH, Berlin, Germany) with the Al Kα excitation radiation (1486.7 eV). The surface concentration of elements was determined from survey spectra considering the photoelectron cross-sections for elements. The XPS Ni 2p_3/2_ and N 1s lines were approximated using Gaussian/Lorentzian functions after subtraction of Shirley’s spectral background. X-ray absorption spectroscopy measurements were performed on the 8 beamline channel of the VEPP-3 storage ring at the Budker Institute of Nuclear Physics (Novosibirsk, Russia). The incident energy was selected using the ⟨111⟩ reflection from a double Si crystal monochromator. During the measurements, the storage ring mode corresponded to the energy of 2 GeV and current of 70–140 mA. Ni K-edge EXAFS spectra were measured with a step of 2 eV in the range of 800 eV above the absorption edge at room temperature in the standard transmission mode by means of ionization chambers filled with Ar/He and Xe as monitoring and final detectors, respectively. EXAFS data extraction (pre-edge subtraction, spline background removal) was performed using the VIPER 10.17 software. Radial pair distribution functions around the Ni atoms were obtained through the Fourier transformation of k^1^- and k^3^-weighted EXAFS functions across the ranges of photoelectron wave numbers k = 2.0 to 11.0 Å^−1^. The local environment of the Ni atom (interatomic distances (Ri), coordination numbers (Ni), and distance mean square deviations from thermal motion and static disorder of the absorbing and scattering atoms, known as Debye–Waller factors (σ^2^)) was modeled in the EXCURVE software. Ni K-edge XANES spectra were measured with a step of 0.5 eV in the range of 150 eV before and 100 eV above the absorption edge. The modeling of XANES spectra was carried out in the FEFF 9.0 [55] and IFEFFIT (ATHENA) software. All the spectra were normalized on an absorption jump.

### 5.3. Catalytic Measurements

The set-up for the catalytic decomposition of formic acid is described in detail elsewhere [56]. Briefly, argon passed through a glass container-bubbler filled with liquid formic acid, which saturated the inert gas. After additional dilution with argon, the concentration of formic acid vapor reached 2.5 vol% and the total gas flow rate was equal to 1.1 mL s^−1^. The catalyst was placed over a piece of quartz wool into a glass fixed-bed reactor located in the furnace and was pretreated in the same formic acid/Ar flow at 350 °C for 30 min before each experiment to reduce Ni and stabilize the catalyst. The products of the decomposition reaction (CO, CO_2_ and H_2_) were analyzed by a Chromos GC-1000 gas chromatograph (Chromos Engineering, Dzerzhinsk, Russia). Gas concentrations were determined with a standard deviation of 5%. Details of the gas chromatographic analysis, conversion and specific reaction rate calculations are described in the Supporting Information file.

### 5.4. DFT Calculations

The calculations were performed using the long-range-corrected hybrid Perdew-Burke-Ernzerhof (LC-ωPBE) functional [57]. Owing to the exact asymptote of the exchange potential, this functional performs remarkably well for a wide range of molecular properties and, in particular, for bond lengths and long-range charge transfer. The lacv3p triple-zeta basis set, where the inner core electrons are replaced by pseudopotentials, was used for Ni atomic orbitals and all-electron split-valence basis set with inclusion of polarization functions (6–31 g*) was applied in the case of light elements (C and N). The basis sets were chosen from a comparison of calculated and experimental data for nickel dimer, nickelocene and nickel porphyrin. The LC-ωPBE/lacv3p method gives the preferred Ni_2_ triplet state with a Ni-Ni bond length of 2.0999 Å, which is in good agreement with the experimental value of 2.1545(4) Å [58]. The used calculation method with the 6–31 g* basis set for light elements correctly predicts the energy preference for the triplet state of nickelocene and the singlet state of nickel porphyrin. The resulting average Ni-C bond length in nickelocene is 2.178 Å and the Ni-N bond length in nickel porphyrin is 1.951 Å. The values perfectly agree with the corresponding experimental bond lengths of 2.185 Å [59] and 1.951(2) Å [42], respectively. 

The pseudo-spectral method was used to simplify the Coulomb and exchange operators as implemented in the Jaguar software package (Jaguar, version 10.3, Schrödinger, Inc., New York, NY, USA, 2019) [60]. The integrals were evaluated fully analytically for the models with six nickel atoms in order to achieve the convergence. The geometry of the models was optimized in all possible spin states without any symmetry restrictions by the analytical gradient method up to the root mean squared change of the density matrix elements less than 5.0 × 10^−6^. The local minimum was confirmed by the zero number of imaginary frequencies. The binding energy of the Ni atom to the support was calculated as E^bin^ = E^tot^(sup) + E^tot^(Ni) − E^tot^(model), where the members correspond to the total energy of the support in the ground state, the energy of one Ni atom in the triplet state and the total energy of the model in the ground state, respectively. A positive value of E^bin^ indicates the energy gain due to the Ni attachment. 

## Figures and Tables

**Table 1:** Structural data for the 6Ni/CN sample obtained from the fitting of EXAFS spectrum (N_i_—coordination number; R_i_—interatomic distance, 2σ_i_^2^—Debye–Waller factor, F_i_—a factor representing statistical error of the fitting).

Sample	Shell	N_i_	R_i_ (Å)	Ratio of Coordination Sites (Ni-N_4_/Ni-O)	2σ^2^·10^−2^ (Å^2^)	F_i_ *
6Ni/CN	Ni-N	4 (fixed)	1.96 ± 0.01	30/70	1.4 ± 0.4	1.7
	Ni–O	5.5 ± 0.4	2.09 ± 0.01			
	Ni–O–Ni	0.8 ± 0.3	3.11 ± 0.02		2.5 ± 0.7	

* $F_{i}=\sum_{i}^{N}w_{i}^{2}{\left(\chi_{i}^{exp}\left(k\right)-\chi_{i}^{th}\left(k\right)\right)}^{2},w_{i}=\frac{k_{i}^{n}}{\sum_{i}^{N}k_{i}^{n}\vee \chi_{j}^{exp}\left(k\right)\vee }$.

**Table 2:** The XPS-derived surface concentration of the main elements in 6Ni/CN and 6Ni/C.

Sample	Ni, at%	C, at%	N, at%	O, at%
6Ni/C	0.10 ± 0.01	94.3 ± 4.7	-	5.6 ± 0.3
6Ni/CN	0.70 ± 0.04	85.3 ± 4.3	5.5 ± 0.3	8.5 ± 0.4

**Table 3:** The content of different Ni coordination centers in 6Ni/CN obtained by the linear combination fitting of the Ni K-edge XANES spectrum.

Sample	Linear Combination			R-Factor
	1Ni/CN (Ni-N_4_)	Ni-c	Ni-e	
**6Ni/CN**	25 ± 7	31 ± 5	44 ± 5	0.0008

## Data Availability

Not applicable.

## Supplementary Materials

The following supporting information can be downloaded at: https://www.mdpi.com/article/10.3390/nano13030545/s1, Figure S1: HAADF/STEM image of 1Ni/C and the size distribution of nanoparticles; Figure S2: High resolution TEM images of 6Ni/C and 6Ni/CN after the reaction; Figure S3: HAADF/STEM image of 6Ni/CN after the reaction and EDX spectrum from this area; Figure S4: Raman spectra of 1Ni/C, 6Ni/C, 1Ni/CN and 6NiCN; Figure S5: Comparison of experimental 1Ni/CN Ni K-edge spectrum with theoretical spectra plotted for flat and curved clusters.
