# Supplementary material for: Highly Dispersed Ni on Nitrogen-Doped Carbon for Stable and Selective Hydrogen Generation from Gaseous Formic Acid
Source: Nanomaterials (Basel). 2023 Jan 29;13(3):545. doi: 10.3390/nano13030545 (PMC9921425; doi:10.3390/nano13030545)
Supplement: Supplementary file 1 [file nanomaterials-13-00545-s001.zip › nanomaterials-2140476-supplementary.pdf]

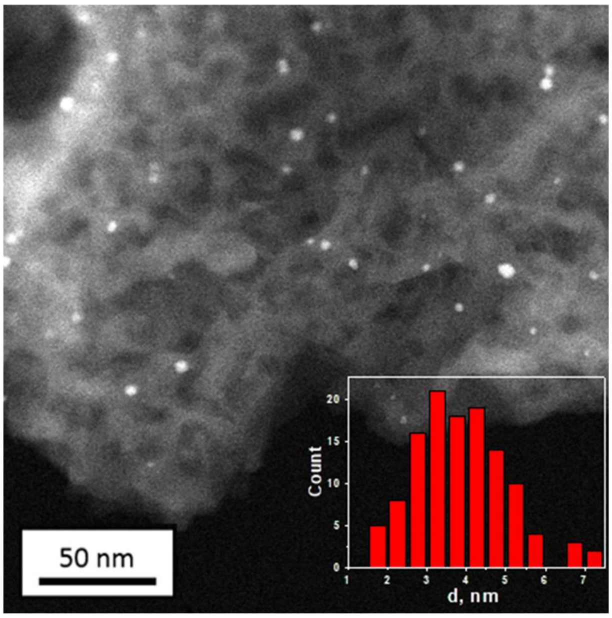


**Figure S1.** HAADF/STEM image of 1Ni/C. Inset presents the size distribution of nanoparticles (bright dots in the image).

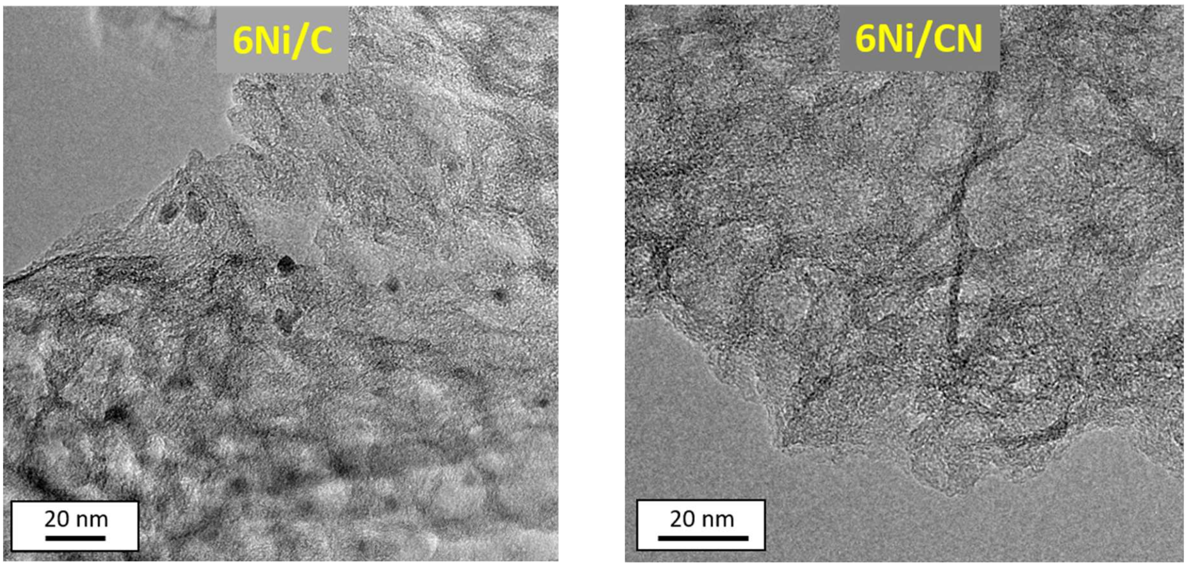


(**a**) (**b**)

**Figure S2.** Transmission electron microscopy images of (**a**) 6Ni/C and (**b**) 6Ni/CN nanomaterials after the catalytic reaction.

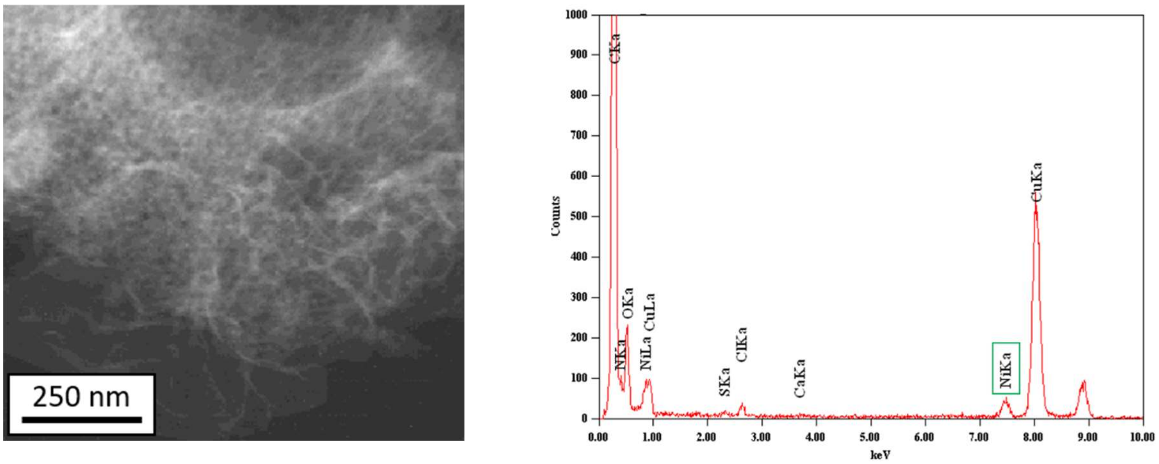


(**a**) (**b**)

**Figure S3.** (**a**) HAADF/STEM images of the 6Ni/CN nanomaterial after the catalytic reaction and (**b**) EDX spectrum from this area. The high intensity of the O K$\alpha$ line is due to the contribution of the oxidized surface of the copper grid.

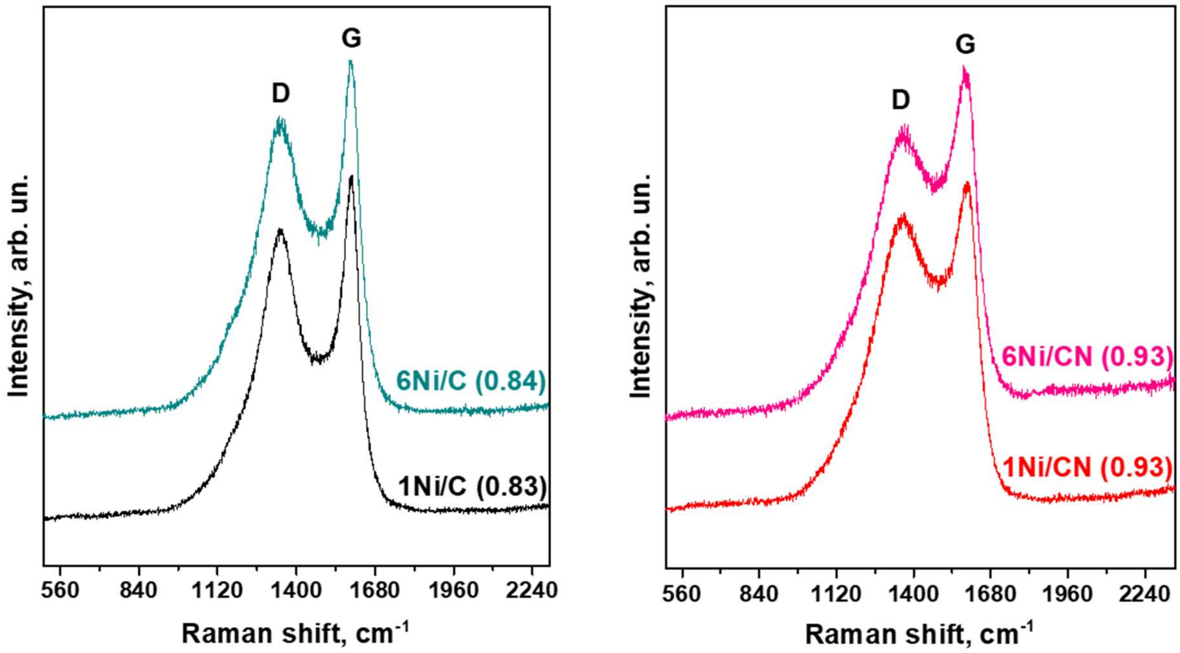


(**a**) (**b**)

**Figure S4.** Raman spectra of (**a**) 1Ni/C and 6Ni/CN and (**b**) 1Ni/CN and 6Ni/CN nanomaterials after the catalytic reaction. The $I_D/I_G$ value is given in brackets.

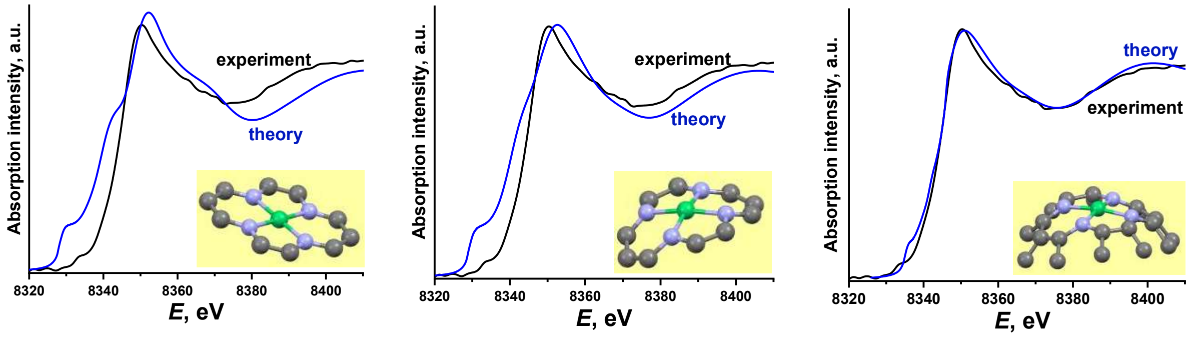


(**a**) (**b**) (**c**)

**Figure S5.** Comparison of experimental XANES Ni K-edge spectrum of the starting 1Ni/CN nanomaterial with theoretical spectra plotted for clusters shown in insets: (**a**) flat Ni-$N_4C_{10}$ with Ni–N bond length of 1.87 Å, distorted (**b**) Ni-$N_4C_{10}$ and (**c**) Ni-$N_4C_{20}$ with an average Ni–N bond length of 1.96 Å. Green, blue, and grey balls correspond to Ni, N, and C atoms, respectively.

## Details of the gas chromatographic analysis and related calculations

The products were analyzed by a gas chromatograph (Chromos GC-1000) equipped with two thermal conductivity detectors and packed columns filled with CaA molecular sieves and HayeSep-Q porous polymer. For separation of the products, heating of the columns from 40 to 210 °C was performed within 10 min and then the temperature was kept stable for 20 min. The calibration was performed using gas mixtures with known concentrations.

The conversion of formic acid (X) was determined as the ratio of the sum of the obtained concentrations of CO and $H_2$ to the initial concentration of formic acid:

$$X = \frac{C_{CO} + C_{H_2}}{C_{HCOOH}} \times 100\%,$$

From the obtained conversion values, the specific reaction rates (W) were calculated, related to the mass of Ni in the catalyst:

$$W = \frac{X \times C_{HCOOH} \times V_f \times N_A}{22{,}400 \times m_{Ni}} \quad \left[\frac{\text{molecule}}{s * g_{Ni}}\right],$$

where $C_{HCOOH}$ is the initial formic acid concentration in the Ar flow, $V_f$ is the total flow rate (mL $s^{-1}$), $N_A$ is the Avogadro constant (is equal to $6.022 \cdot 10^{23}$ molecule $mol^{-1}$), $m_{Ni}$ is the Ni mass in the catalyst sample (g), and 22,400 is the volume of one mole of gas at 273 K and 1 atm (mL $mol^{-1}$).
